# Enhancing the Mechanical and Frost Resistance Properties of Sustainable Concrete Using Fired Pumice Aggregates

**DOI:** 10.3390/ma18174191

**Published:** 2025-09-06

**Authors:** Mahiro Hokazono, Momoka Ijichi, Takato Tsuboguchi, Kentaro Yasui

**Affiliations:** 1 Department of Civil and Environmental Engineering, School of Environment and Society, Institute of Science Tokyo, 2-12-1 Ookayama, Meguro-ku, Tokyo 152-8550, Japan; hokazono.m.2290@m.isct.ac.jp; 2 Construction Coordination Division, Fukuoka International Airport Co., Ltd., 782-1 Shimousui, Hakata-ku, Fukuoka 812-0003, Japan; momoka-ijichi@fukuoka-airport.co.jp; 3 Graduate School of Science and Technology, Kumamoto University, 2-39-1 Kurokami, Chuo-ku, Kumamoto 860-8555, Japan; 250d8316@st.kumamoto-u.ac.jp; 4 Department of Urban Environmental Design and Engineering, National Institute of Technology, Kagoshima College, 1460-1 Shinko, Hayato-cho, Kirishima 899-5193, Japan

**Keywords:** pumice, fired pumice, lightweight concrete, sand substitute, sustainable construction

## Abstract

This study addresses the problem of pumice deposits in the southern Kyushu region, which can cause landslides during heavy rainfall. To reduce this hazard, it is important to expand pumice applications and promote its use before disaster events occur. Among construction materials, this study explores the possibility of using pumice as a concrete aggregate, considering the global shortage of natural aggregates. Because of the low strength and difficulty of use, pumice must be fired to improve its properties. In our experiment, it was fired at 1000 or 1100 °C, and the performance of the resulting concretes was compared. Concrete incorporating pumice fired at 1100 °C achieved a maximum compressive strength of 54.6 N/mm^2^ with an increase in the amount of cement, whereas concrete with pumice fired at 1000 °C remained within the 20–24 N/mm^2^ range even when the amount of cement was increased. This difference arises because pumice has a lower strength than the cement paste, leading to material failure. Furthermore, freeze–thaw tests showed that concrete made with pumice fired at 1100 °C was resistant to frost damage. These results suggest that pumice fired at 1100 °C has an excellent potential as a sustainable building material.

## 1. Introduction

**Figure 1:**
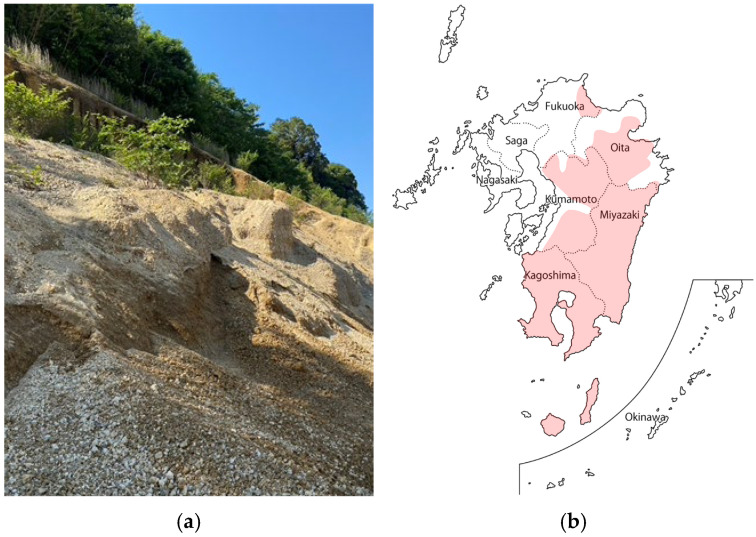
Pumice outcrops and distribution areas: (**a**) Photograph of a pumice outcrops distribution; (**b**) Distribution zones of special soils containing pumice in Kyushu [2].

Japan has 111 active volcanoes [1], of which 17 are located in Kyushu, where pumice, a type of volcanic debris, is widely deposited. Based on data from the Ministry of Agriculture, Forestry and Fisheries [2], in Kyushu, zones designated as special soils include Bora (pumice), Shirasu, and Akahoya, among others, all of which are highly susceptible to erosion (Figure 1) [3,4].

Furthermore, southern Kyushu, including Kagoshima and Miyazaki prefectures, experiences a high frequency of typhoons [5] and extremely heavy rainfall, making the region highly prone to landslides. Consequently, substantial pumice deposits are often washed away by these events, causing severe damage to residential areas as well as to agriculture, forestry, and fisheries sectors. However, despite this pressing issue, the budget allocated for countermeasures against special soils has been declining [6].

Given these circumstances, mining pumice and utilizing its unique properties effectively are both necessary and desirable for sustainable development.

In Japan, pumice is primarily used for soil drainage improvement [7] and as a gardening material [8]. Its porous nature provides excellent water retention and drainage properties; however, the amount of pumice used for these purposes remains very small compared with the amount that accumulates, and the demand is currently low.

Concrete aggregates are essential for infrastructure development; however, transportation costs often exceed their value, making the use of locally available aggregates desirable. Therefore, aggregates are generally mined near concrete manufacturing sites. In Japan, there is a growing movement to prohibit the mining of river gravel, river sand, sea gravel, and sea sand owing to the depletion of natural aggregates [9] and the need for environmental conservation. Consequently, crushed limestone and crushed sand are now widely used as alternatives. However, this type of limestone is also a raw material for steel, cement, and concrete aggregates, and stricter mining conditions have emerged owing to rising demand as well as environmental and landscape regulations. Therefore, it is predicted that limestone reserves will be depleted by 2050 [10]. Furthermore, the global sand shortage has become so severe that it is now referred to as the “sand crisis” [11,12]. In this context, use of pumice as an alternative fine and coarse aggregate for concrete could help alleviate the sand shortage problem and contribute to disaster mitigation through pumice mining.

Pumice can be used in concrete in two main techniques: (i) as a partial substitute for cement or a precursor after crushing and (ii) directly as a coarse or fine aggregate [13].

Kabay et al. [14] reported that replacing 10–20% of cement with powdered pumice reduced 7 days strength compared with cement alone, although the difference in the 180 days strength was minimal.

The use of pumice as a precursor in geopolymers has also been investigated. Hamid et al. [15] proposed a combination of fly ash and pumice as precursors and reported that even 100% pumice could achieve a compressive strength of 31 N/mm^2^, 28 days after thermal curing at 60 °C for 6 days. The above study suggests that pumice also exhibits a certain degree of alkali activation. Furthermore, Zayad et al. [16] developed a hybrid geopolymer concrete using 70% fine pumice powder and 30% cement, which achieved a compressive strength of 32 N/mm^2^ at 90 days, thereby highlighting the potential to reduce cement usage.

Kabay et al. [17] found that replacing 50–100% of fine aggregates (1–4 mm) with pumice aggregates of the same size decreased the strength as the replacement ratio increased. Cavaleri et al. [18] demonstrated that pumice is an effective substitute for artificial lightweight aggregates in structural wall materials. Hossain et al. [19] revealed that slump was reduced by approximately 22–38% by replacing 50–100% of natural aggregate with pumice. Parhizkar et al. [20] reported that pumice aggregates led to significant increases in drying shrinkage as the concrete aged, due to moisture loss. Previous studies also reported significantly higher water absorption with pumice aggregates [21,22,23], and polymer coating has been proposed as a countermeasure. Bideci et al. [24] reported that the use of polymer-coated pumice aggregate (PCPA) reduced water absorption by ~10%, whereas the compressive strength decreased by more than 50%. Tuncer et al. [25] showed that the 28 days and 90 days compressive strengths using PCPA were 28.1 to 11.8 N/mm^2^ and from 32.2 to 18.0 N/mm^2^, respectively, as the replacement ratio increased from 0 to 100%.

These studies indicate that crushed pumice can be used as a cement admixture or geopolymer precursor; however, its low strength as an aggregate, owing to the presence of fine pores, has limited its use. To overcome this issue, the present study fired pumice study to improve its strength and adjusted its particle size for concrete applications. Concrete specimens were then prepared to evaluate compressive strength and Young’s modulus tests. In addition, the decrease in frost resistance due to the porosity and high water absorption rates of pumice was examined.

Previous studies have not systematically compared the effects of different firing temperatures on pumice aggregates in concrete, or clearly demonstrated the relationship between mechanical properties and frost resistance. The findings of this study therefore contribute to the effective use of pumice mined in the region and the mitigation of sand shortages through the use of sustainable concrete aggregates.

The remainder of this paper is organized as follows: Section 2 explains the firing method and physical properties of pumice. Section 3 compares the compressive strength, Young’s modulus, and freeze–thaw test results of fired pumice concrete with those of previous studies. Section 4 summarizes the test results and presents future prospects.

## 2. Materials and Methods

### 2.1. Firing Method and Physical Properties of Pumice Aggregates

#### 2.1.1. Pumice

**Figure 2:**
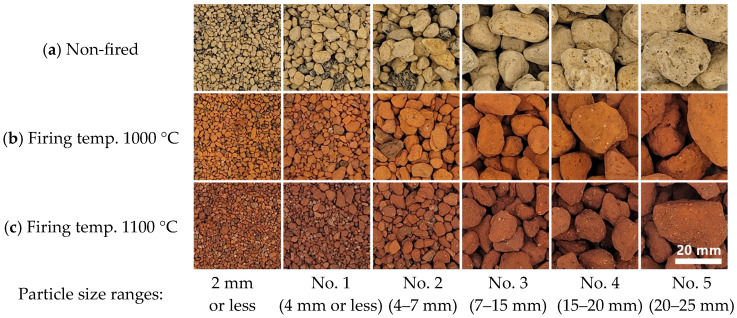
Appearance of pumice by grain size: (**a**) Non-fired; (**b**) Firing temp. 1000 °C; (**c**) Firing temp. 1100 °C.

In this study, pumice from Miyakonojo City, Miyazaki Prefecture, was used. This material, locally known as “bora,” is one of the pyroclastic products of an eruption in the Kirishima volcanic belt approximately 4600 years ago. It is characterized by numerous fine pores, which provide excellent water absorption and permeability. Pumice classified by miners’ company is divided into particle size ranges: 2 mm or less, No. 1 (4 mm or less), No. 2 (4–7 mm), No. 3 (7–15 mm), No. 4 (15–20 mm), and No. 5 (20–25 mm), as shown in Figure 2. These are commercially available in home improvement stores, and the classified pumice was used in this study. Prior to use, pumice was fired at 1000 °C or 1100 °C, and the fired samples are also shown in Figure 2.

#### 2.1.2. Firing Method

**Figure 3:**
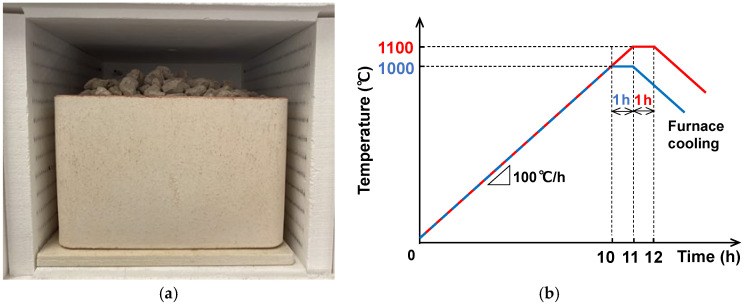
Firing method: (**a**) Pumice in an alumina container; (**b**) Changes in temperature and time during firing.

The pumice was fired in an electric muffle furnace (FUW252PA, Toyo Seisakusho Kaisha, Ltd., Kashiwa, Japan). Samples of each particle size range were placed in an alumina container with inner dimensions of 245 × 245 × 180 mm, as shown in Figure 3a, and fired. The changes in temperature and time during firing are shown in Figure 3b. Specifically, the temperature was first increased from room temperature at a rate of 100 °C/h, and after reaching a maximum temperature of 1100 °C, it was maintained at this temperature for 1 h. The pumice was then allowed to cool in the furnace.

The structure of pumice becomes denser upon firing, thereby improving the strength of the aggregate. After firing, the pumice stone was cooled to room temperature, packed into a cup with an inner diameter of 30 mm and a height of 20 mm, and embedded in resin. The resin cylinder containing the hardened pumice stone was polished using a lapping machine to expose the pumice surface. Vickers hardness (JIS R 1610 [26]) was then measured by recording the width of the depression when a Vickers indenter was pressed against the cylinders. The Vickers hardness of non-fired pumice was 14 HV, whereas pumice fired at maximum temperatures of 900, 1000, and 1100 °C were 33, 171, and 476 HV, respectively [27]. The increase in hardness was remarkable when fired at 1000 and 1100 °C. This behavior is consistent with structural ceramics such as bricks and tiles, which also become denser upon firing, and display improved mechanical properties [28]. Specifically, it is considered that moisture and impurities between pumice particles are removed during firing, leading to particle bonding and shrinkage.

#### 2.1.3. Microstructural Observations and Crystal Structure Analysis of Pumice

**Figure 4:**
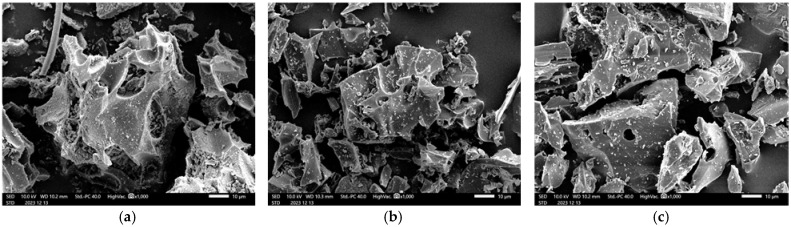
SEM images of pumice: (**a**) Non-fired; (**b**) Firing at 1000 °C; (**c**) Firing at 1100 °C.

To examine the microstructural changes caused by firing in the pumice, we analyzed pumice that had been crushed and passed through a 150 µm sieve. Microstructural observations were conducted using a scanning electron microscope (SEM; JSM-IT200, JEOL Ltd., Akishima, Japan) with an acceleration voltage of 10 kV. To make the pumice conductive, the samples were coated with a 30 nm thick layer of Au using sputtering. SEM images of non-fired and fired pumice are displayed in Figure 4. Pumice stones were randomly sampled for each firing condition, and consistent results were obtained. Pores of 10–20 µm were observed in the non-fired pumice, whereas pores of ~5 µm were observed in the fired pumice, indicating shrinkage due to firing. However, no clear differences could be confirmed between the pumice samples fired at 1000 °C and 1100 °C from the SEM images.

**Figure 5:**
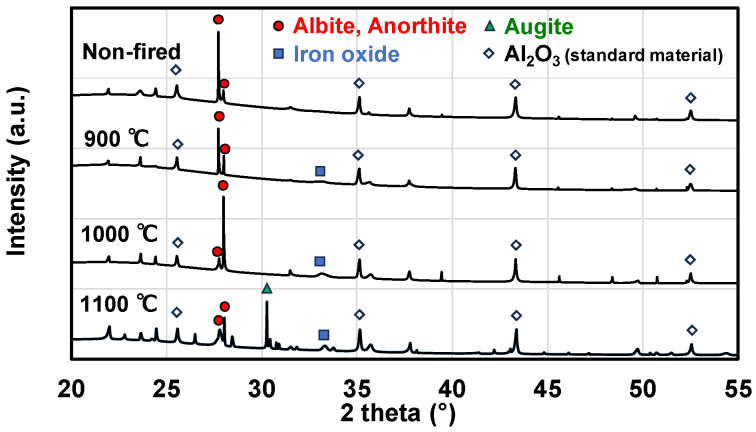
Crystal structure of pumice.

X-ray diffraction (Smart Lab, Rigaku Holdings Corporation, Akishima, Japan) was performed to analyze the crystal structure of pumice, and the analysis results are shown in Figure 5. The measurement conditions were as follows: Cu tube: 40 kV, 30 mA; measurement range: 20–55°, step width: 0.01°/step; measurement speed: 1.4°/min; entrance slit: 1/6°. Pumice samples were mixed with 10 mass% α-Al_2_O_3_ as a standard material, and the obtained diffraction intensity pattern was corrected based on the intensity of the α-Al_2_O_3_ peak.

Peaks corresponding to albite (NaAlSi_3_O_8_) and anorthite (CaAl_2_Si_2_O_8_) were observed regardless of the firing temperature. With increasing firing temperature, peaks of augite ((Ca, Mg, Fe)_2_Si_2_O_6_) and iron oxide (Fe_2_O_3_) were observed, indicating partial crystallization via sintering. The color change of pumice to reddish-brown after firing is attributed to the influence of iron oxide.

#### 2.1.4. Grain Size Adjustment of Pumice for Use as Aggregate for Concrete

The mined pumice is classified into grain sizes of 2 mm or less and Nos. 1 to 5 to meet domestic demand in Japan; however, it cannot be used as an aggregate for concrete in its original state. Therefore, a sieving test was conducted to determine the particle size distribution, and multiple sizes of pumice were mixed to prepare fine and coarse aggregates. The standard range of particle sizes (JIS A 5005 [29]) specified by the Japan Society of Civil Engineers was used as a reference.

**Figure 6:**
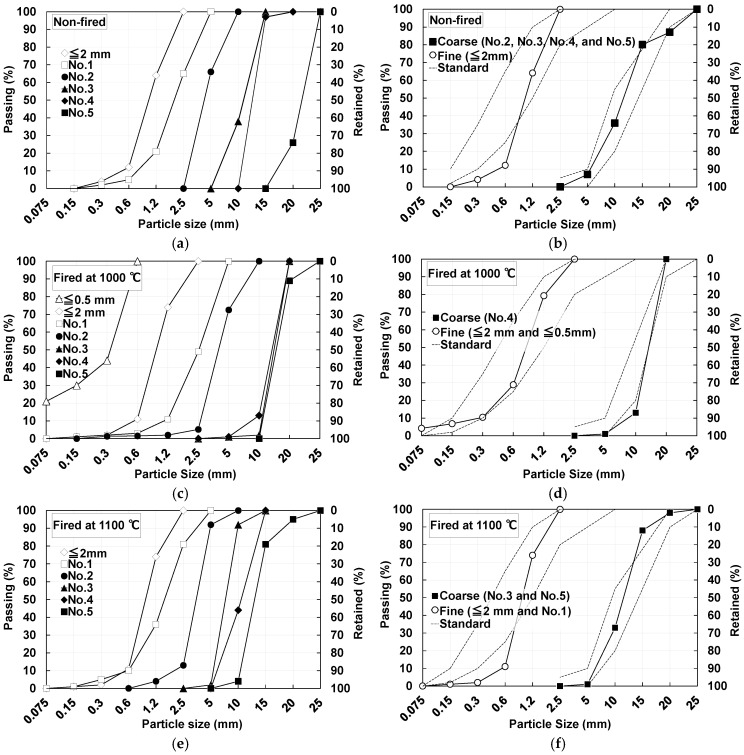
Particle size distribution of classified pumice and that of pumice after adjustment for concrete aggregate: (**a**) Classified non-fired pumice; (**b**) Aggregates prepared with non-fired pumice; (**c**) Classified pumice fired at 1000 °C pumice; (**d**) Aggregates prepared with pumice fired at 1000 °C pumice; (**e**) Classified pumice fired at 1100 °C pumice; (**f**) Aggregates prepared with pumice fired at 1100 °C pumice.

Figure 6a shows the particle size distribution of non-fired pumice. Fine aggregate of 2 mm or less was used directly from non-fired pumice. Coarse aggregate was prepared by mixing Nos. 2, 3, 4, and 5 in mass ratios of 1:5:1:2 (Figure 6b). Figure 6c shows the particle size distribution of pumice fired at a maximum temperature of 1000 °C. Fine aggregate was prepared by mixing pumice ≤ 2 mm with pumice ≤ 0.5 mm in a mass ratio of 4:1; whereas coarse aggregate consisted of No. 4 (15–20 mm) (Figure 6d). Figure 6e shows the particle size distribution of pumice fired at a maximum temperature of 1100 °C. Fine aggregate was prepared by mixing pumice ≤ 2 mm with No. 1 in a mass ratio of 8:1 and coarse aggregate was prepared by mixing Nos. 3 and 5 in a mass ratio of 1:2 (Figure 6f).

#### 2.1.5. Density, Absorption, Bulk Density, and Solid Content of Pumice

The density and absorption of pumice aggregates adjusted to a particle size distribution suitable for concrete aggregates were measured in accordance with JIS A 1135 [30]. Oven-dried aggregates are usually immersed in water for 24 h to absorb water before density measurement. However, because pumice is porous, 24 h was considered insufficient for water to penetrate the internal pores. Therefore, the test was conducted after 10 d of water absorption. Bulk density and solid content were measured in accordance with JIS A 1104 [31].

The physical properties of the pumice are listed in Table 1. The density of the oven-dried, non-fired, coarse aggregate was 0.59 g/cm^3^, and it increased to 0.89 g/cm^3^ when fired at 1000 °C. Therefore, if the viscosity is low, it may float in water. However, this value increased to 1.54 g/cm^3^ when fired at 1100 °C due to pore shrinkage as a result of firing. In contrast, the non-fired fine aggregates had a density of 1.58 g/cm^3^, which further increased with firing temperature. The higher density of the fine aggregate is attributed to the smaller particle size, which leaves fewer voids. Consequently, the density in the saturated surface-dry condition of the fine aggregates was higher than that of the coarse aggregates. The water absorption of non-fired pumice was 95%, and this value decreases as the firing temperature increases. Fine aggregates consistently showed lower absorption than coarse aggregates. Bulk density remained below 1 kg/L for all aggregates except the fine aggregate fired at 1100 °C, which was still lower than the bulk density of the natural aggregate (1.5–1.8 kg/L) [32,33]. The solid content of pumice ranged between 57 and 60% for both non-fired and fired aggregates, which exceeds the requirements for crushed stone (56%) for concrete and crushed sand (54%) in concrete [29]. This result is attributed to the non-angular shape of the aggregates and the ability to optimize packing by mixing large and small particles.

### 2.2. Preparation of Pumice Concrete

The mixing proportions of the concretes prepared in this study are listed in Table 2. The materials used were as follows: tap water (W) and ordinary Portland cement (C) (Tokuyama Corporation, Shunan, Japan), non-fired or fired pumice as fine aggregates (S) and coarse aggregates (G) in oven-dry condition, and an air entraining water-reducing agent (Type I) (Sika Pozzolith 15L, Sika Japan Ltd., Tokyo, Japan) was used as the water reducer (Ad), added at 1.5 wt% of cement.

The water–cement ratio (W/C) of the concrete with non-fired pumice was 55% and 491 kg/m^3^ of cement was used.

When pumice fired at 1000 °C was used, the unit water amount was fixed at 300 kg/m^3^ and the W/C was varied in the range of 65–80% by increasing the amount of cement. However, when the W/C was 60%, the cement and pumice absorbed water, and fluidity was not achieved; therefore, the unit water amount was increased to 350 kg/m^3^, and the amounts of the fine and coarse aggregates were adjusted accordingly. This mixing proportion is described in Section 3, but the high water content caused floating of coarse aggregates in fresh concrete, and this mix did not exhibit strength; so, it is reported in parentheses for reference.

Furthermore, when pumice fired at 1100 °C was used, the unit water content was fixed at 247 kg/m^3^, and the cement content was increased to adjust the W/C in the range of 40–60%.

Fresh concrete was prepared in accordance with JIS A 1138 [34] in a laboratory maintained at 20 °C and 60% humidity. A vibrating mixer (OM-10E, Tiger Chiyoda Materials Co., Ltd., Tokyo, Japan) was used for mixing. Cement, fine aggregate, and coarse aggregate were first mixed for 2 min, after which water mixed with a water reducer was poured in and mixed for another 2 min. The rotation speed was 120 rpm during the entire process.

The mixed concrete was cast in accordance with JIS A 1132 [35]. For the compression and Young’s modulus tests, samples were packed into a cylindrical mold with a diameter of 100 mm and height of 200 mm, whereas for the freeze–thaw test, they were packed into a square mold with a cross section of 100 mm × 100 mm and a length of 400 mm. After compacting the specimens using a table vibrator (TS-450 × 600, Mikasa Sangyo Co., Ltd., Tokyo, Japan), they were cured in water at 20 °C until the specified age.

### 2.3. Physical Property Tests of Concrete

#### 2.3.1. Slump Test

Slump test was conducted for fresh concrete in accordance with JIS A1101 [36]. The mixed concrete was packed into a cone-shaped mold in three layers and the cone was gently removed. The change in the height of the concrete was measured as the slump value.

#### 2.3.2. Compression Test and Young’s Modulus Measurement

**Figure 7:**
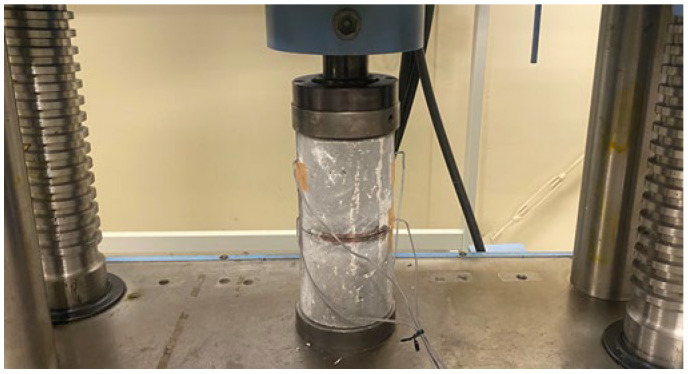
Compression test and Young’s modulus measurement.

The compressive strength of the concrete was measured in accordance with JIS A 1108 [37]. The specimens that had been cured underwater to the specified age were loaded using a universal testing machine (ACA-200D-F3, Maekawa Testing Machine MFG. Co., Ltd., Tokyo, Japan), as shown in Figure 7, and the maximum load at the time of failure was measured. The compressive strength was calculated by dividing the measured maximum load by the cross-sectional area of each specimen.

Young’s modulus measurement was conducted in accordance with JIS A 1149 [38]. Two strain gauges were attached in the loading direction and two in the circumferential direction to a 28 d specimen, and the load was applied in the same manner as in the compression test. The load (converted to stress by dividing by the cross-sectional area) and strain were measured during the test, and the Young’s modulus was calculated using the following equation:

(1) $$E_{c}=\frac{\sigma_{1}-\sigma_{2}}{\varepsilon_{1}-\varepsilon_{2}}\;\left(N/{mm}^{2}\right)$$

where

$E_{c}$: Young’s modulus (N/mm^2^)
$\sigma_{1}$: Stress (N/mm^2^) equivalent to 1/3 of the maximum stress (compressive strength)
$\sigma_{2}$: Stress (N/mm^2^) at which the strain is 50 μ
$\varepsilon_{1}$: Strain corresponding to the stress equivalent to 1/3 of the maximum stress
$\varepsilon_{2}$: 50 μ

#### 2.3.3. Freezing and Thawing Test

Freeze–thaw tests for concrete are generally conducted in accordance with ASTM C 666 [39] and JIS A 1148 [40]. However, lightweight concrete using highly absorptive aggregates such as pumice typically exhibits lower freeze–thaw resistance than ordinary concrete. Therefore, conventional test conditions are considered too severe for evaluating the frost resistance of lightweight concrete [41].

**Figure 8:**
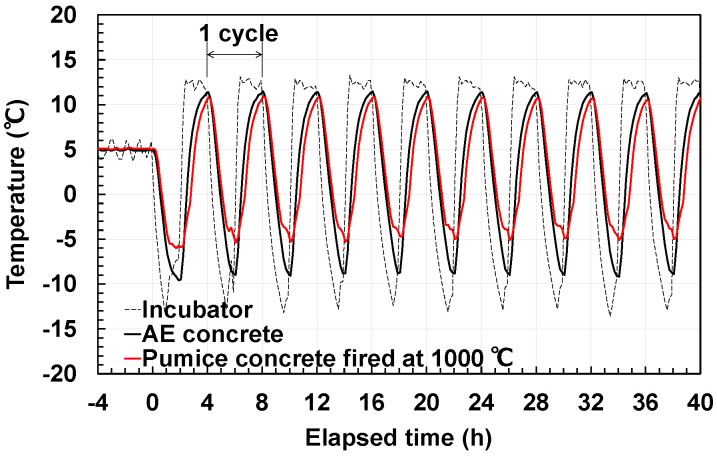
Temperature changes during freeze–thaw cycles.

In this study, we adopted the method from Uematsu et al. [41], where they assumed actual environmental conditions and evaluated the frost resistance of fired pumice concrete. We used a 100 mm × 100 mm × 400 mm square column specimen aged 28 d and conducted the test under environmental conditions representative of a slightly cold region. In this test, an incubator (NRB-14A, Nihon Freezer Co., Ltd., Tokyo, Japan) was used, and 300 cycles of accelerated testing were conducted in the temperature range of −5–10 °C for 4 h per cycle (Figure 8).

To evaluate the frost resistance, specimens were removed at specific intervals within the first 36 cycles, and the ultrasonic velocity was measured using an ultrasonic measurement device (Ultracon-170, M.K.C Korea, Seoul, South Korea). The relative ultrasonic propagation velocity (UPV) was calculated using Equation (2) [42]. For concrete using pumice fired at 1000 °C, the first resonance frequency (Hz) was measured, in addition to the ultrasonic velocity for each specified cycle using a dynamic Young’s modulus measurement device (MIN-011-0-12, Marui & Co., LTD., Daito, Japan). The relative dynamic elastic modulus was calculated using Equation (3) [43].

(2) $$R_{c}=\frac{{V_{n}}^{2}}{{V_{0}}^{2}}\times 100\;\left(\%\right)$$

where

$R_{c}$: Relative UPV after n cycles (%)
$V_{n}$: UPV after n cycles (m/s)
$V_{0}:$ UPV at 0 cycles (m/s)

(3) $$P_{n}=\frac{{f_{n}}^{2}}{{f_{0}}^{2}}\times 100\;\left(\%\right)$$

where

$P_{n}$: Relative dynamic modulus of elastic after n cycles (%)
$f_{n}$: Primary resonance frequency of flexural vibration after n cycles (Hz)
$f_{0}$: Primary resonance frequency of flexural vibration at 0 cycles (Hz)

## 3. Results and Discussion

### 3.1. Slump of Fresh Concrete and Aggregate Distribution

**Figure 9:**
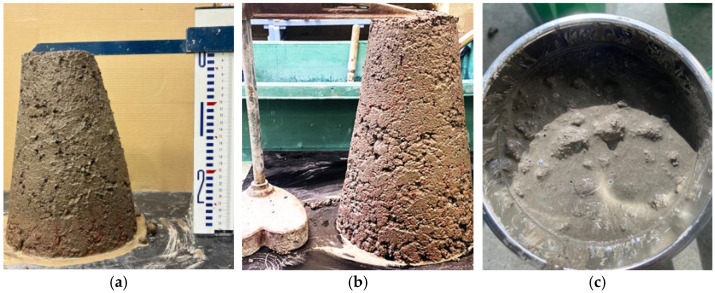
Fluidity of fresh concrete: (**a**) Slump test status of concrete mixed with aggregate fired at 1000 °C (W/C = 80%, Slump = 3.0 cm); (**b**) Slump test status of concrete mixed with aggregate fired at 1100 °C (W/C = 55%, Slump = 1.0 cm); (**c**) Concrete flowing inside the mold during vibration compaction.

Figure 9a,b show the photographs of the slump test of fresh concrete, and Table 3 lists the corresponding slump values. The values obtained in this study were 1.5 cm, 0–3 cm, and 1–2 cm for non-fired pumice concrete, 1000 °C fired pumice concrete, and 1100 °C fired pumice concrete, respectively. These results are consistent with a previous study [18], which reported that pumice concrete has a low slump value. No material separation was observed after tapping, and good fluidity was observed after vibration, as shown in Figure 9c. This is because the surface-dry density of fired pumice is 1.20–2.26 g/cm^3^, which is significantly smaller than that of general aggregates (2.6–2.8 g/cm^3^), and it is considered that it flowed together with the cement paste.

**Figure 10:**
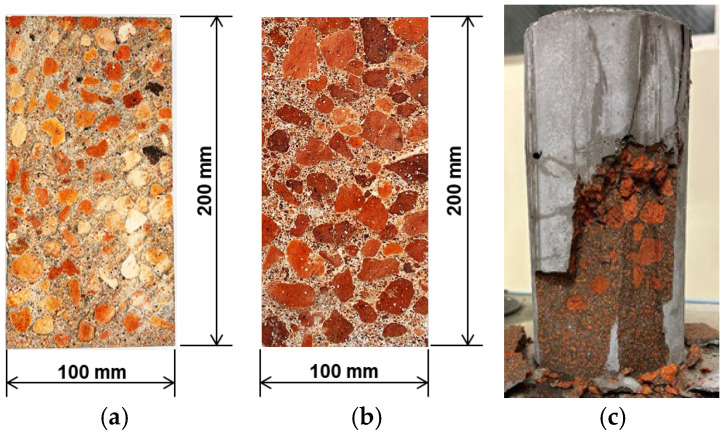
Cut surface of the specimens: (**a**) Concrete mixed with aggregate fired at 1000 °C; (**b**) Concrete mixed with aggregate fired at 1100 °C; (**c**) Coarse pumice aggregate floated to the surface, reducing the strength.

Figure 10 shows the aggregate distribution after cutting the hardened concrete specimens. Although the density of fired pumice is low, no aggregate floating was observed in the mix used in this experiment, and it can be inferred from the cut surface that the viscosity of the cement paste was sufficient. However, when the unit water content was increased to 350 kg/m^3^ using the mix in Table 2, the slump reached 22.0 cm (Table 3), the coarse pumice aggregates floated during vibration compaction (Figure 10c), and the compressive strength of the hardened concrete was significantly reduced. Therefore, even with a low slump value, fluidity is not a problem. If greater fluidity is required, it is more appropriate to increase the amount of water-reducing agent and viscosity rather than the water content.

### 3.2. Compressive Strength and Young’s Modulus

**Figure 11:**
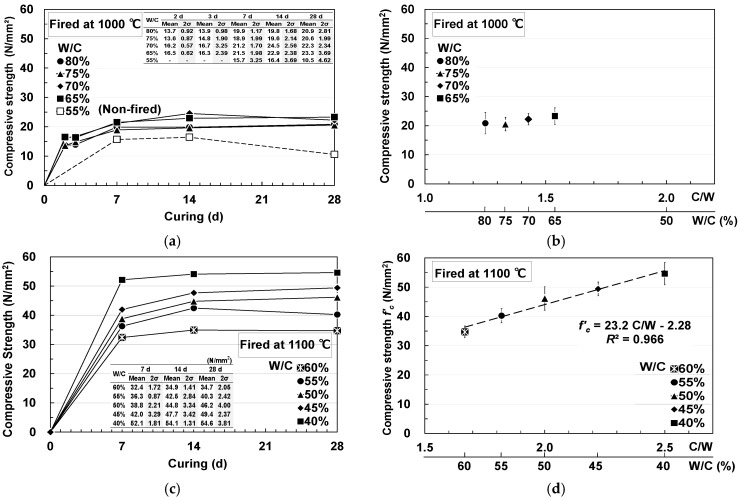
Compressive strength of pumice concrete (error bars = 2σ): (**a**) Change in the compressive strength of 1000 °C fired pumice concrete; (**b**) Relationship between the 28 d compressive strength of 1000 °C fired pumice concrete and C/W; (**c**) Change in the compressive strength of 1100 °C fired pumice concrete; (**d**) Relationship between the 28 d compressive strength of 1100 °C fired pumice concrete and C/W.

Figure 11a shows the relationship between W/C and compressive strength over time for pumice concrete fired at 1000 °C. As W/C decreases at 2 d, the compressive strength increases; however, after 7 d, the compressive strength converges to 18–24 N/mm^2^ regardless of W/C, with no further improvement in strength. In other words, even though the unit cement content was increased from 375 to 462 kg/m^3^, the 28 d strength improved by only 5 N/mm^2^. In contrast, the maximum compressive strength of non-fired pumice concrete was 16.4 N/mm^2^, which is comparable to the values reported in previous studies [44,45,46].

Figure 11b shows the relationship between the compressive strength and cement-to-water ratio (C/W) for pumice concrete fired at 1000 °C. Unlike concrete with normal aggregates, no clear linearity between the C/W and compressive strength was observed.

Figure 11c shows the relationship between W/C and compressive strength over time for pumice concrete fired at 1100 °C. At 28 d, a high compressive strength of 54.6 N/mm^2^ was achieved at a W/C of 40%, and the compressive strength increased further as W/C decreased.

Figure 11d illustrates the relationship between compressive strength and C/W for pumice concrete calcined at 1100 °C. A linear relationship was observed, allowing both W/C and C/W to be easily calculated up to approximately 50 N/mm^2^.

**Figure 12:**
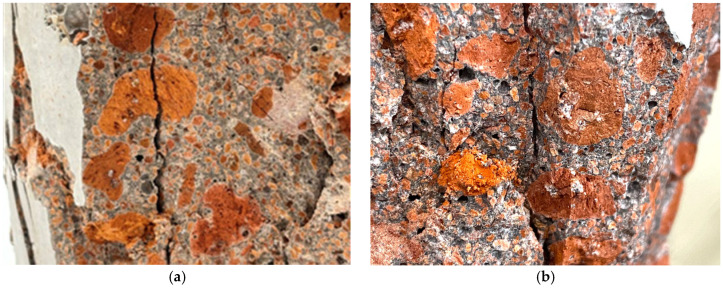
Fracture cross section after compression test: (**a**) Pumice fired at 1000 °C has a lower strength than cement paste; therefore, cracks are progressing through the pumice. (**b**) Because the pumice fired at 1100 °C is stronger than the cement paste, cracks develop at the interface between the pumice and the paste.

Furthermore, fracture surface observations after compression test showed that, for pumice fired at 1000 °C, the cement paste strength exceeded the pumice strength as C/W increased, and material failure of the pumice occurred first (Figure 12a). Therefore, pumice fired at 1000 °C can be used as an aggregate for concrete with a nominal strength of approximately 18 N/mm^2^, but it is unsuitable for higher nominal strength applications. However, when pumice fired at 1100 °C was used, the strength of the pumice was higher than that of the cement paste, and interfacial failure occurred at the interface between the cement paste and pumice (Figure 12b). Therefore, when pumice fired at 1100 °C is used, the strength of the concrete can be increased by adjusting the cement content—an advantage not achievable with non-fired pumice. Tuncer et al. [25] reported 28 d strengths of 28.1 N/mm^2^ for concrete prepared with pumice (4–8 mm and 8–16 mm) and crushed sand (0–4 mm) with a cement content of 450 kg/m^3^ and W/C of 50%. However, in this study, pumice concrete fired at 1100 °C exceeded that strength using pumice alone.

**Figure 13:**
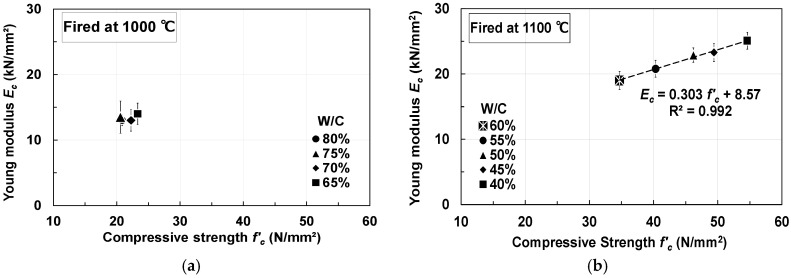
Relationship between compressive strength and Young’s modulus (error bars = 2σ): (**a**) Pumice concrete fired at 1000 °C; (**b**) Pumice concrete fired at 1100 °C.

Figure 13 shows the relationship between compressive strength and Young’s modulus. The compressive strength of pumice concrete fired at 1000 °C was 20–24 N/mm^2^, and the Young’s modulus remained low at 13–14 kN/mm^2^. However, the Young’s modulus of pumice concrete fired at 1100 °C increased with compressive strength in the range of 19.0–25.0 kN/mm^2^ (C/W = 1.67–2.50), and a linear relationship was observed between them. This improvement is attributed to the increase in concrete density caused by the increase in cement content.

In addition, the Young’s modulus of concrete fired at 1100 °C was lower than that of ordinary concrete but significantly higher than that of non-fired pumice (approximately 8.0–15.0 kN/mm^2^ [47]). These results suggest that firing pumice enhances both compressive strength and Young’s modulus. The Poisson’s ratio remained constant at 0.2.

### 3.3. Freeze Resistance

**Figure 14:**
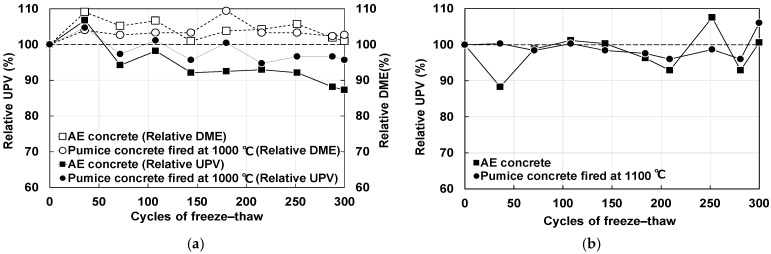
Freeze resistance of pumice concrete: (**a**) Changes in relative ultrasonic velocity and relative dynamic modulus of elasticity of concrete fired at 1000 °C; (**b**) Changes in the relative ultrasonic velocity of concrete fired at 1100 °C.

Figure 14a shows the relationship between relative UPV, relative dynamic modulus of elasticity (DME), and number of cycles for pumice concrete fired at 1000 °C. A high correlation between relative UPV and relative DME has been reported in previous studies [42,48]. For comparison, the results for Air Entrained (AE) concrete made with normal aggregate and an AE agent are also shown in the figure. The relative UPV and relative DME represent the average of three measurements; error bars are not shown because 2σ was approximately 1% for each case.

The relative UPV of pumice concrete fired at 1000 °C fluctuated around 100% throughout the 300 freeze–thaw cycles, with no significant decrease. Similarly, the relative DME was approximately 100% up to 180 cycles, after which it showed a slight reduction.

Figure 14b shows the change in the relative UPV of pumice concrete fired at 1100 °C. It also remained close to 100% after 300 cycles. If the relative DME is ≥ 90% after 300 freeze–thaw cycles, the material is considered to have excellent resistance. Pumice concrete fired at 1000 °C meets this requirement, and since relative DME and relative UPV can be evaluated in the same manner, concrete fired at 1100 °C can also be regarded as frost resistant. However, since concrete fired at 1000 °C exhibited a decline after 180 cycles, further evaluation is necessary before its use in cold regions.

**Figure 15:**
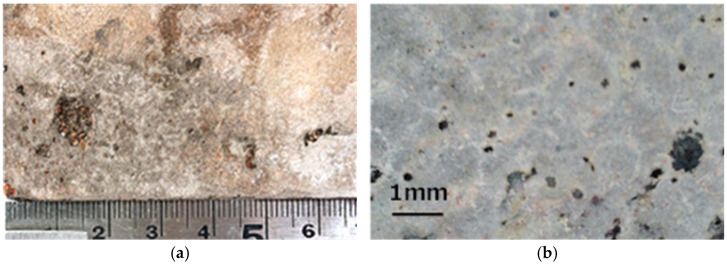
Concrete surface after 300 cycles: (**a**) Pumice concrete fired at 1000 °C; (**b**) Pumice concrete fired at 1100 °C.

Figure 15 shows the surface of the specimens after 300 freeze–thaw cycles. Several pop-outs were observed on the surface of the pumice concrete fired at 1000 °C. In contrast, the surface of the pumice concrete fired at 1100 °C remained intact and maintained a healthy state. This result shows that resistance to expansive pressure caused by the porous structure of pumice depends on the firing temperature. Specifically, pumice fired at 1000 °C could not withstand the expansive pressure, whereas that fired at 1100 °C exhibited sufficient resistance. Another possible reason for this improved result is the lower water absorption rate of the pumice fired at 1100 °C. However, because the environmental conditions for this test were limited (freeze–thaw cycle: −5 °C to 10 °C), detailed prior consideration is required before using it in colder regions with more severe conditions.

Besheli et al. [49] prepared concrete in which cement was replaced with a combination of 15% lime and 15% pumice, thereby improving its frost resistance. This suggests that pumice is effective not only as an aggregate but also as a supplementary cementitious material.

The temperature change inside the specimens during the freeze–thaw test (Figure 8) indicates that fired pumice concrete tends to exhibit a lower heat transfer rate than regular concrete. This phenomenon may be attributed to the decrease in thermal conductivity caused by the large amount of porous pumice included. However, further verification of this effect is required.

In this study, we assumed that pumice with low strength can be fired to improve its strength and subsequently used as an aggregate for concrete. In the experiments, pumice was fired in an electric furnace at 1000 °C or 1100 °C. However, large-scale production of precast products or concrete structures would require continuous firing in a rotary kiln. If pumice can be mined in areas where aggregates cannot be extracted or on remote islands, it could facilitate concrete production. Furthermore, pumice is lightweight, allowing the transport of large amounts at once. Therefore, the findings of this study highlight the effective use of pumice, which can be mined locally, as a sustainable aggregate for concrete. However, strengthening pumice through firing requires significant thermal energy input, which likely increases the environmental burdens in terms of CO_2_ emissions and fuel consumption. Therefore, it is necessary to reduce CO_2_ emissions by utilizing the exhaust heat from the kiln to dry the pumice and by replacing fuels derived from wood pellets and waste plastics with coke and liquefied natural gas (LNG).

## 4. Conclusions

Concrete was prepared using fired pumice to ensure its effective utilization and alleviate the sand shortage problem. The main findings are summarized below.

1. Firing pumice caused shrinkage, which increased its density and reduced the pores inside it, thereby increasing its strength as an aggregate.
2. Concrete with a good balance of large and small particles can be produced by adjust-ing the particle size of the fired pumice.
3. The compressive strength of concrete using pumice fired at 1000 °C converged to 20–23 N/mm^2^. As the pumice strength was lower than that of cement paste, material fail-ure occurred. This suggests that it can be used for concrete with a nominal strength of approximately 18 N/mm^2^; however, the benefit of firing at 1000 °C is minimal because of the high energy required for firing and the low strength achieved.
4. The compressive strength of pumice concrete fired at 1100 °C can be increased by ad-justing W/C, with a maximum strength of 54.6 N/mm^2^. The corresponding Young’s modulus is in the range of 19.0–25.0 kN/mm^2^, lower than that of conventional con-crete. However, it can be used as a general-purpose aggregate.
5. The frost resistance of fired pumice concrete was the same for both 1000 °C and 1100 °C firing conditions, as determined by relative UPV and relative DME after 300 freeze–thaw cycles. However, pop-outs were observed on the concrete surfaces using 1000 °C fired pumice, requiring careful consideration before use. By contrast, concrete with 1100 °C fired pumice exhibited excellent frost resistance.

This study contributes to mitigating the problem of landslides caused by heavy rains in the southern Kyushu region through effective pumice utilization while also addressing global concerns regarding sand depletion in construction. This study clarifies the influence of firing temperature on the compressive strength and frost resistance of pumice concrete and demonstrates its potential as a sustainable general-purpose aggregate, enabling effective utilizing of locally-mined resources while reducing environmental impact.

The limitations of the study include the following:

1. Optimization of firing conditions: Only two firing temperatures, 1000 °C and 1100 °C, were analyzed. However, to achieve target nominal strengths (30 N/mm^2^, 40 N/mm^2^, 50 N/mm^2^, etc.) while reducing firing energy, intermediate firing temperatures such as 1050 °C and shorter firing times should be considered. Machine learning techniques can be valuable in this regard. For instance, Jabin et al. [50] created concrete incorporating recycled nylon fiber and volcanic pumice powder in cement and used a random forest algorithm to predict its properties with high accuracy. By training models with data on firing temperature, cement amount, and unit water content, it may be possible to predict strength and frost resistance, and to identify the key influencing factors.
2. Applicable range of freeze–thaw tests: The freeze–thaw cycles were limited to a temperature range of −5 to 10 °C. A wider range of temperature conditions are necessary for use in extremely cold regions in the future.
3. Investigation of the effects of shrinkage: The porous nature of pumice may cause the absorbed water to escape after the concrete hardens, resulting in shrinkage [19]. This study did not examine this phenomenon in detail; therefore, further research is required to clarify the water absorption characteristics and their effects.

Possible future prospects include the following:

1. Improvement of efficiency in the production of fired pumice concrete: Because on-site mix adjustments are needed to match the water absorption rate of the pumice, production is currently most feasible for small precast products such as road curbs and gutter covers. Research into improving production efficiency will broaden the scope of pumice applications.
2. Contribution to a recycling-oriented society: Crushed pumice can be used as an admixture to reduce the amount of cement used. We aim to develop cement-free construction materials by producing geopolymer products using crushed pumice as a precursor and fired pumice as an aggregate. Additionally, by combining pumice with other recycled aggregates, we aim to create a resource-circulating society.

## Figures and Tables

**Table 1:** Physical properties of pumice.

Firing Temp.	Aggregate	Density in Oven-Dry Condition (g/cm^3^)	Density in Saturated Surface-Dry Condition (g/cm^3^)	Absorption (%)	Bulk Density (kg/L)	Solid Content (%)
Non-fired	Coarse	0.59	1.20	95.0	0.40	58
	Fine	1.58	1.91	20.8	0.90	57
1000 °C	Coarse	0.89	1.43	60.4	0.54	60
	Fine	1.60	1.98	23.9	0.92	57
1100 °C	Coarse	1.54	1.86	20.9	0.85	58
	Fine	2.08	2.26	9.45	1.12	59

**Table 2:** Mixing proportions of the concrete.

Firing Temp.	W/C (%)	s/a (%)	Quantity of Material per Unit Volume (kg/m^3^)				
			W	C	S	G	Ad
Non-fired	55	47	270	491	427	180	7.4
1000 °C	80		300	375	437	274	5.6
	75			400	431	270	6.0
	70			429	424	266	6.4
	65			462	417	261	6.9
	(60)		(350)	(583)	(350)	(220)	(8.8)
1100 °C	60		247	412	609	508	6.2
	55			449	597	499	6.7
	50			494	584	487	7.4
	45			549	566	473	8.2
	40			618	545	455	9.3

**Table 3:** Slump of fresh concrete.

Firing Temp.	W/C (%)	Slump (cm)
Non-fired	55	1.5
1000 °C	80	3.0
	75	2.0
	70	2.0
	65	0.0
	(60)	(22.0)
1100 °C	60	2.0
	55	1.0
	50	1.0
	45	1.0
	40	3.5

## Data Availability

The original contributions presented in this study are included in the article. Further inquiries can be directed to the corresponding author.
